# ANCA-associated vasculitis following sotatercept initiation in a patient with heritable pulmonary arterial hypertension and previously silent eosinophilic granulomatosis with polyangiitis: a case report

**DOI:** 10.1093/ehjcr/ytag025

**Published:** 2026-01-27

**Authors:** Angelo Laconi, Silvia Mancini, Federica Decandia, Pierluigi Merella, Gavino Casu

**Affiliations:** Clinical and Interventional Cardiology, AOU Sassari, Sassari 07100, Italy; Clinical and Interventional Cardiology, AOU Sassari, Sassari 07100, Italy; Clinical and Interventional Cardiology, AOU Sassari, Sassari 07100, Italy; Clinical and Interventional Cardiology, AOU Sassari, Sassari 07100, Italy; Clinical and Interventional Cardiology, AOU Sassari, Sassari 07100, Italy; Department of Medicine, Surgery and Pharmacy, University of Sassari, Sassari 07100, Italy

**Keywords:** Pulmonary arterial hypertension, Sotatercept, Eosinophilic granulomatosis with polyangiitis, MPO-ANCA vasculitis, Eosinophilia, Activin-TGF-β signalling, Right-ventricular failure, Case-report

## Abstract

**Background:**

Pulmonary arterial hypertension (PAH) is a progressive disease characterized by increased pulmonary vascular resistance and right ventricular failure. Sotatercept, a novel activin receptor ligand trap, has demonstrated promising haemodynamic benefits in PAH treatment. Eosinophilic granulomatosis with polyangiitis (EGPA) is an extremely rare, Th2-driven, small-vessel vasculitis, and its overlap with PAH is scarcely reported.

**Case summary:**

We report the case of a woman with heritable PAH initially stabilized with oral therapy. In 2024, after further clinical decline, subcutaneous treprostinil was initiated. Subsequently, sotatercept was added, resulting in brief clinical improvement. Within weeks, however, the patient developed severe eosinophilia and exhibited laboratory and histopathological evidence of p-ANCA-positive necrotizing vasculitis, accompanied by renal and hepatocellular dysfunction.

**Discussion:**

This case suggests that sotatercept’s modulation of the TGF-β pathway may unmask latent autoimmune diseases such as EGPA in predisposed individuals. Although the temporal relationship between sotatercept initiation and the onset of vasculitis is compelling, both causality and underlying molecular mechanisms remain to be elucidated. Further studies are necessary to understand the potential immunomodulatory mechanisms of sotatercept.

Learning points

- Sotatercept may unmask ANCA-associated vasculitis in patients with PAH and latent EGPA.
- Early, unexplained eosinophilia after Sotatercept warrants prompt autoimmune work-up.
- Routine monitoring after Sotatercept initiation should include eosinophil count and eventually ANCA-titers.

## Background

Pulmonary arterial hypertension (PAH) is a progressive disease characterized by increased pulmonary vascular resistance and right ventricular failure. Sotatercept, a first-in-class activin-signalling modulator, has shown promise in PAH by promoting vascular remodelling and improving hemodynamics.^1^

Eosinophilic granulomatosis with polyangiitis (EGPA) is a rare, Th2-driven, small-vessel necrotizing vasculitis marked by tissue eosinophilia and granulomatous inflammation. One-third of EGPA patients are MPO-ANCA-positive, while HLA-linked immunogenetic susceptibility is well established. Organ damage can be caused by eosinophil infiltration or toxic granule release.^2–6^ EGPA progresses through overlapping phases—an initial allergic stage (asthma, rhinosinusitis), an eosinophilic stage, and a final vasculitic stage—though the sequence and timing often vary between patients.^7^

Coexistence of PAH and EGPA is exceptionally uncommon. We present a case of MPO-ANCA vasculitis that emerged following sotatercept initiation in a patient with heritable PAH and previously clinically silent EGPA.

## Summary figure

**Figure:**
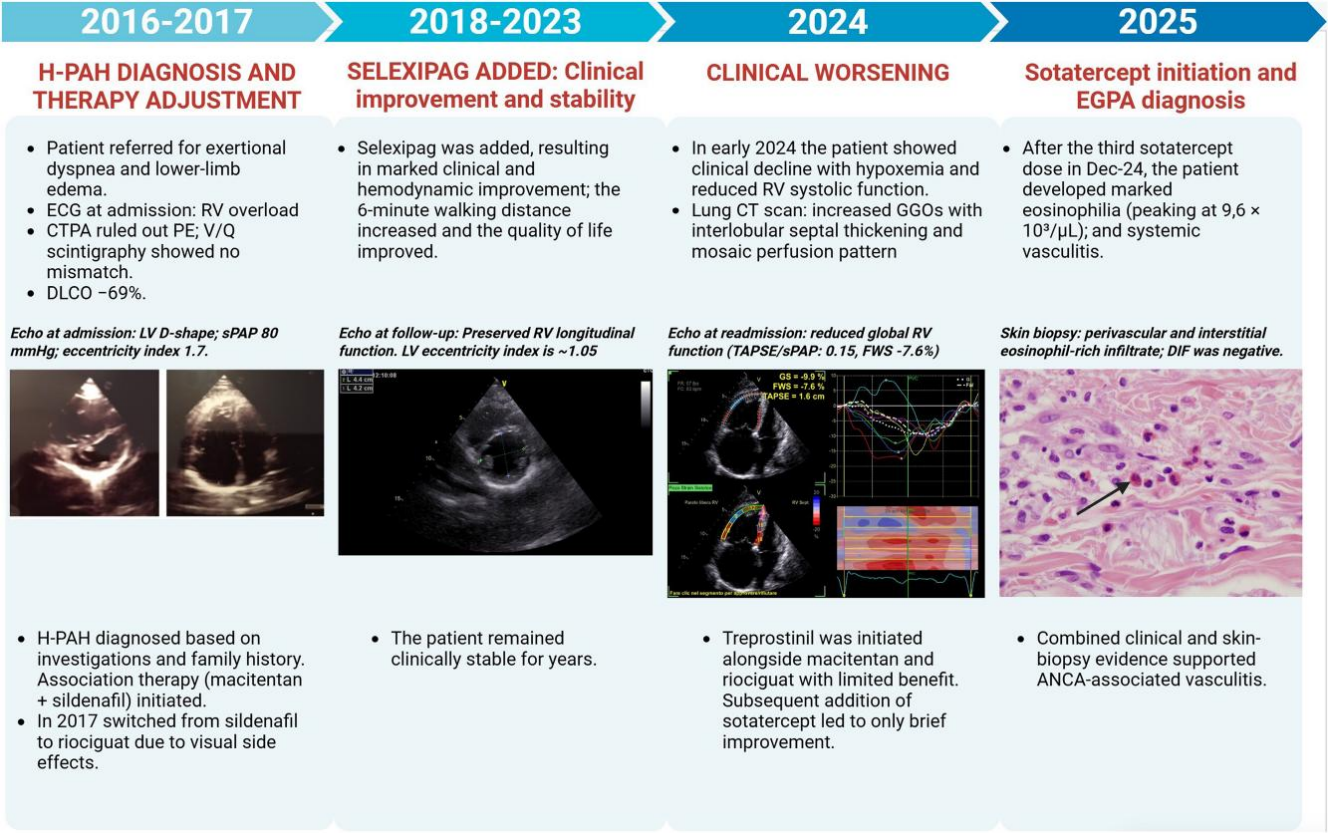


## Case description

The patient, a 48-year-old female with a family history of PAH (mother and maternal uncle), was referred for evaluation of dyspnoea on exertion and lower-limb oedema in 2016. She had previously been physically active and had no significant past medical history. On physical examination, accentuation of the second heart sound was evident, while the electrocardiogram showed signs of right ventricular (RV) overload. She was classified as NYHA class III. The initial echocardiogram revealed a D-shaped left ventricle with an eccentricity index of 1.7 and RV dilatation with mild tricuspid regurgitation. Longitudinal RV systolic function was preserved, while the estimated sPAP was 80 mmHg. A computed tomography pulmonary angiogram (CTPA) ruled out thromboembolic disease but showed centrilobular emphysema and dilatation of the main pulmonary artery. Scintigraphy showed no ventilation–perfusion mismatch. Spirometry and lung diffusion test revealed mild obstructive ventilatory defect with reduced mid-expiratory flows and a reduced DLCO (−69%) [Supplementary material online, *Figure S1*]. The 6-minute walk distance (6MWD) showed reduced exercise tolerance (320 m, 50% of expected). Cardiopulmonary exercise testing (CPET) was suspended at 50 W for exertional dyspnoea, indicating significantly reduced aerobic capacity and poor ventilatory efficiency. Finally, right-heart catheterization (RHC) confirmed pre-capillary pulmonary hypertension, with normal PCWP and elevated mPAP and pulmonary vascular resistance (PVR). Vasoreactivity testing was negative. Although genetic testing was negative for common mutations (KCNK3, BMPR2, CAV1, SMAD9, ATP13A3), based on family history the patient was diagnosed with heritable pulmonary arterial hypertension (H-PAH).

**Figure 1:**
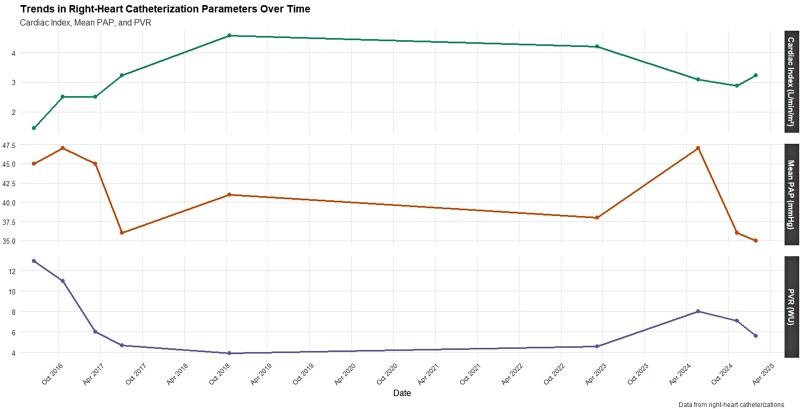
Haemodynamic parameters over-time.

**Figure 2:**
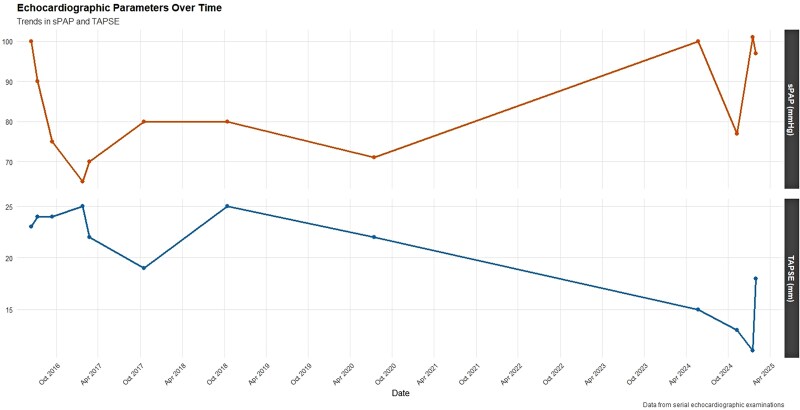
Echocardiographic parameters over-time.

Classified as high risk, the patient was started on upfront dual oral combination therapy with macitentan and sildenafil, with initial clinical stabilization. In July 2017, she reported visual disturbances attributable to sildenafil and was switched to riociguat. At that time, she refused parenteral therapy. At follow-up in June 2018, she showed unsatisfactory clinical and instrumental improvement. Therefore, selexipag was initiated and uptitrated, with tangible clinical improvement. The cardiac index remained above baseline, whereas PVR declined, indicating sustained haemodynamic improvement [*Figure 1*]. Echocardiography showed preserved longitudinal RV systolic function [*Figure 2*]. The 6MWD increased, and a significant improvement in quality of life was observed [Supplementary material online, *Figure S2*]. The patient then remained clinically stable for years.

**Figure 3:**
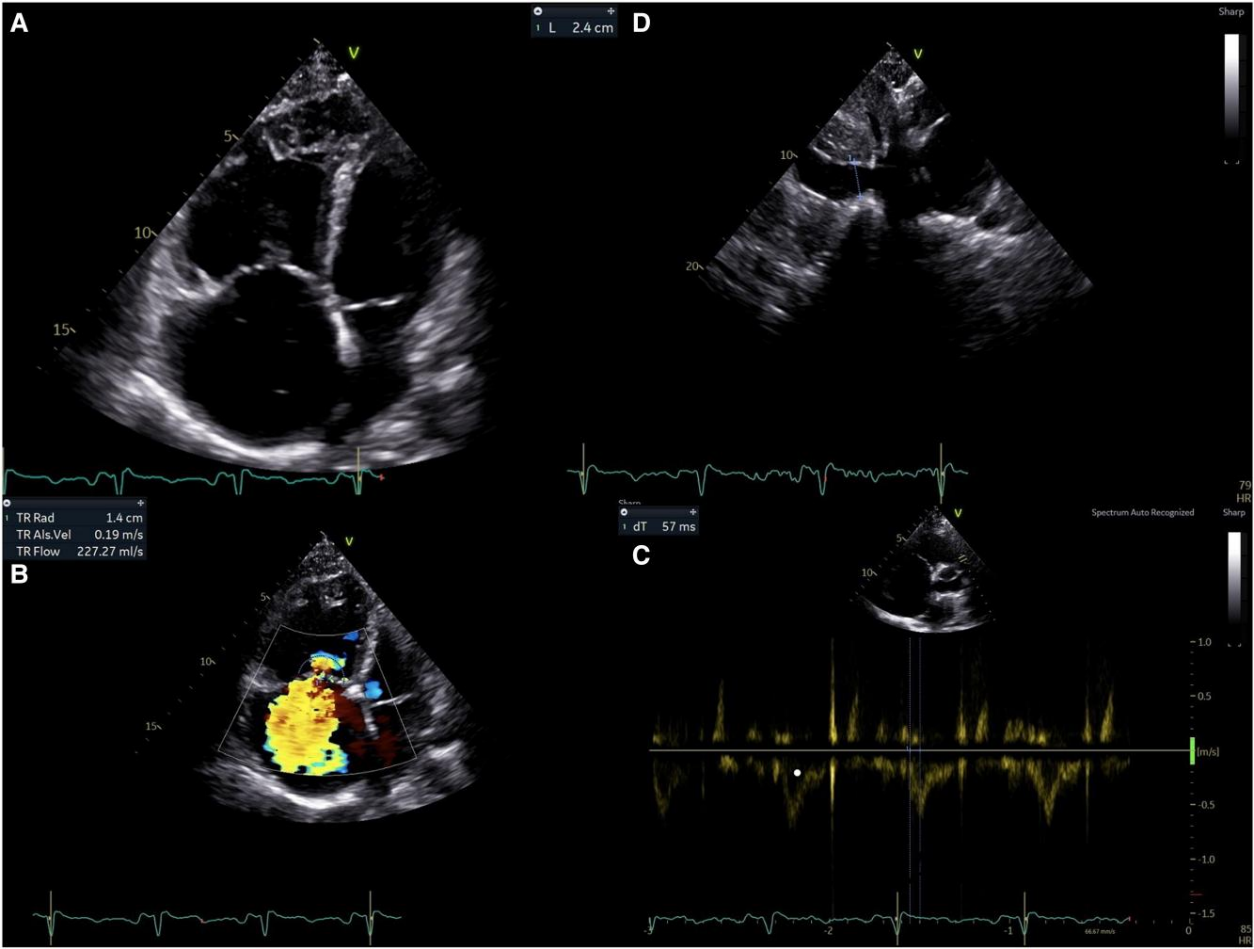
(*A*) Marked right-ventricular dilatation with right-atrial enlargement and interventricular septal flattening, consistent with severe RV pressure overload. (*B*) Severe tricuspid regurgitation with a broad, dense jet filling the right atrium, sPAP estimate of ∼100 mmHg (*C*). Dilated inferior vena cava (≈24 mm) with minimal/absent inspiratory collapse, indicating elevated right-atrial pressure. (*D*) Pulmonary flow (RVOT) pulsed-wave Doppler. Reduced acceleration time (AT ∼57 ms) in the right-ventricular outflow tract supporting severe pulmonary hypertension.

In early 2024, the patient experienced a significant clinical decline, presenting with marked hypoxaemia. Echocardiography revealed severe RV dilatation (RVD1 55 mm) and reduced RV systolic function (FAC 27%, TAPSE 15 mm, FWS −7.6%), with an estimated sPAP of 100 mmHg and a TAPSE/sPAP of 0.15 [*Figure 3*; Supplementary material online, *Table S1*]. HRCT confirmed the known pattern of centrilobular emphysema and showed progressive ground-glass opacities, mosaic perfusion patterns, and enlargement of the pulmonary artery trunk (37.5 mm). RHC revealed an mPAP of 47 mmHg, a PVR of 8 WU, and a CI of 3.08 L/min/m². Parenteral prostanoid therapy was initiated with subcutaneous treprostinil, alongside macitentan and riociguat, with limited clinical benefit.

**Figure 4:**
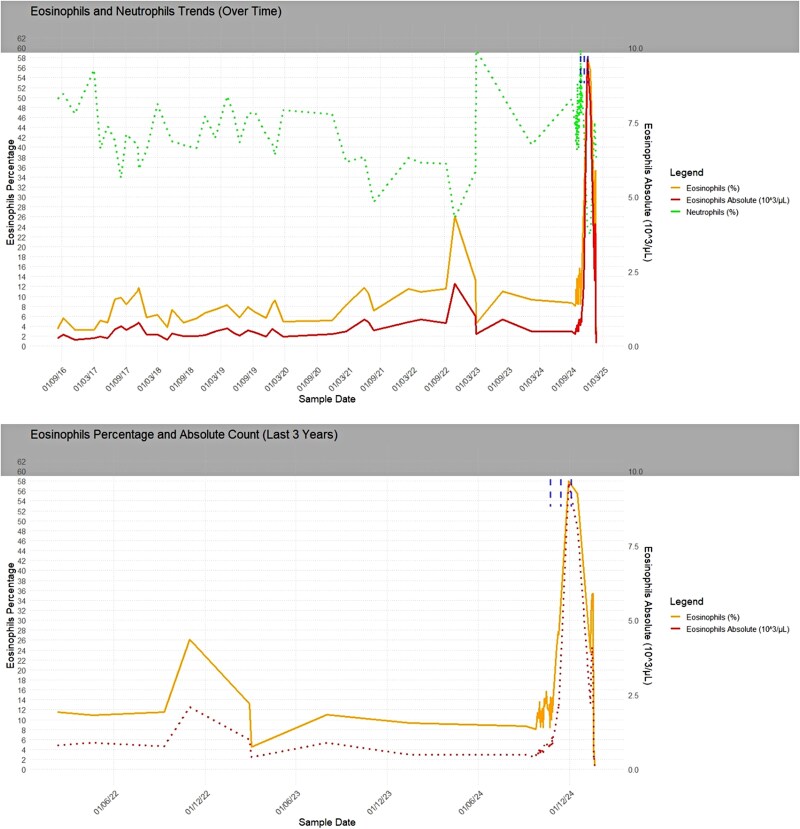
Eosinophilia trend alongside neutrophil fluctuations over-time.

**Figure 5:**
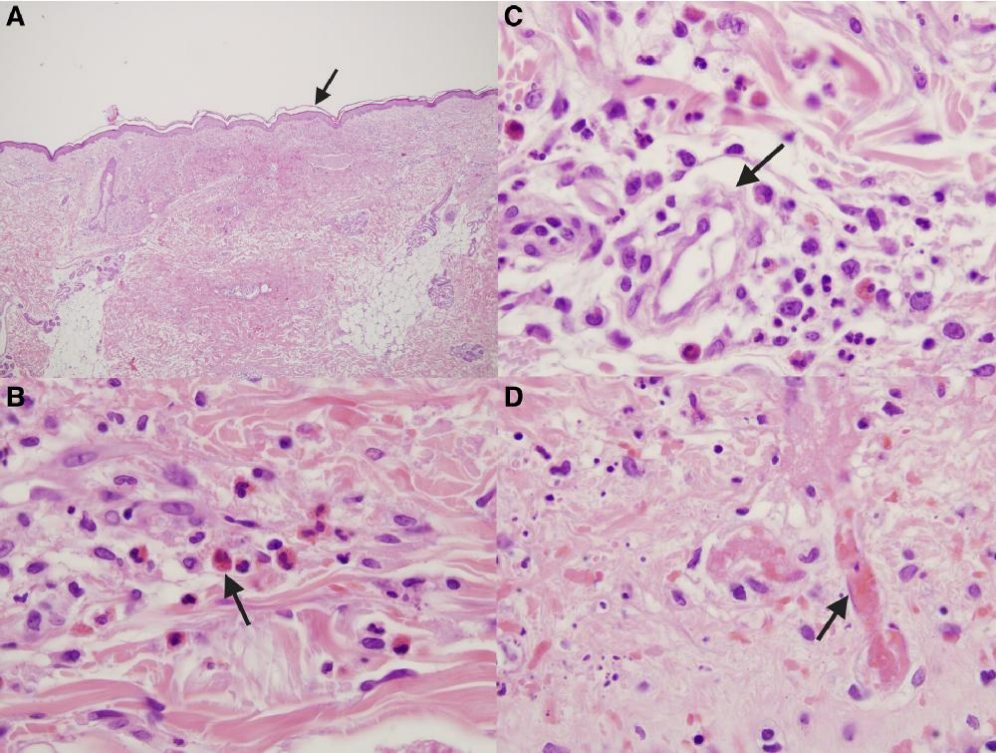
(*A*) Epidermis with focal acantholysis, overlying superficial and mid-dermal perivascular and interstitial inflammatory infiltrates and papillary dermal oedema. (*B*) Infiltrate rich in eosinophils, with foci of eosinophil degranulation. (*C*) Small dermal vessel with inflammatory injury and perivascular eosinophils, compatible with eosinophil-predominant small-vessel vasculitis. (*D*) Fibrinoid necrosis of the vessel wall with superimposed luminal thrombosis of a small-vessel, accompanied by adjacent eosinophils and red-cell extravasation.

Sotatercept was started in October 2024 as add-on therapy for refractory H-PAH. Within weeks, she demonstrated initial clinical improvement; however, laboratory investigations revealed emerging abnormalities. After the third dose in December 2024, marked eosinophilia was observed, with absolute eosinophil counts peaking at 9.6 × 10³/µL and a relative eosinophil percentage of 58%. This hypereosinophilic trend persisted for several days and was accompanied by fluctuations in neutrophil counts [*Figure 4*, Supplementary material online, *Table S3*]. Following these administrations, acute-phase protein levels increased, with evidence of gamma-zone densification (24.2%) on serum electrophoresis. Transaminases initially remained within normal limits. Approximately 2 weeks after the third dose, the patient developed systemic vasculitis with worsening symptoms that prompted hospitalization. Laboratory evaluation in January 2025 revealed p-ANCA positivity, with elevated MPO antibodies (168 IU/mL). Skin biopsy revealed perivascular eosinophilic granulocytes consistent with necrotizing vasculitis [*Figure 5*]. Immunofluorescence studies were negative (IgA, IgM, IgG, C3). Renal involvement became apparent as estimated glomerular filtration rate (eGFR) dropped from 50 to 18 mL/min over one week, while urinary sediment analysis demonstrated casts suggestive of nephropathy. Genetic testing for JAK2, PDGFRA, and FIP1L1 mutations were negative. Peripheral blood smear confirmed hypereosinophilia. Additionally, liver dysfunction emerged, with laboratory findings indicative of severe hepatocellular injury (AST 2609 U/L, ALT 1076 U/L, GGT 190 U/L, alkaline phosphatase 212 U/L, and LDH 2871 U/L). Further hepatological evaluation excluded viral reactivation, with negative serology. Peripheral neuropathy was subsequently confirmed on nerve conduction studies. Echocardiography revealed a markedly dilated RV with severe tricuspid regurgitation and an estimated sPAP of 93 mmHg. Global RV function was depressed, as indicated by the FAC of 24% and FWS of −8.1% [Supplementary material online, *Table S1*]. Heart-failure decompensation was confirmed by a dramatic rise in NT-proBNP levels [Supplementary material online, *Figure S3*].

Given the temporal relationship between sotatercept initiation and the eosinophilic response and vasculitic manifestations, sotatercept was suspected to be the triggering factor and was promptly discontinued. High-dose corticosteroids were started (methylprednisolone 250 mg/day bolus), followed by oral prednisone and rituximab infusion. Eosinophil counts gradually declined, accompanied by clinical improvement and a clear reduction in NT-proBNP levels. Haemodynamic improvement was confirmed by RHC [Supplementary material online, *Table S2*]. Subsequent bedside echocardiography showed progressive improvement in RV function and a reduction in estimated sPAP values. Furthermore, improved respiratory function permitted a reduction in supplemental oxygen delivered via nasal cannula. Despite the sustained improvement, an infectious complication precipitated a rapid clinical decline that progressed to multi-organ failure, and the patient ultimately passed away.

## Discussion

Since 2016, our patient had experienced exertional dyspnoea and an obstructive ventilatory defect—findings initially ascribed solely to heritable PAH but also compatible with a silent eosinophilic-allergic phase of EGPA. Despite mild relative eosinophilia, absolute eosinophil counts remained within limits throughout the 8-year follow-up, rising significantly only once before sotatercept initiation [Supplementary material online, *Table S3*].

Potential alternative aetiologies for hypereosinophilia were systematically assessed. Drug-induced vasculitis was considered; however, the patient had previously tolerated macitentan, riociguat, and treprostinil, while the rise in eosinophils occurred only after the third sotatercept dose, making a delayed reaction to the background regimen unlikely. Notably, no other treatments beyond sotatercept were introduced during that period. Parasitic causes (e.g. *Strongyloides*, *Trichinella*) and fungal infections were excluded by negative serological tests and stool examinations. Primary clonal eosinophilic disorders were improbable in the absence of atypical cells on peripheral smear and with negative PDGFRA, FIP1L1, and JAK2 testing. Whole-body contrast-enhanced CT, esophagogastroduodenoscopy and colonoscopy showed no lesions suspicious for malignancy, while serum tumour markers were within reference limits. Together with p-ANCA/MPO-ANCA positivity and a pauci-immune pattern on direct immunofluorescence, these data support EGPA/ANCA-associated vasculitis as the unifying diagnosis.

Pre-clinical work by Zhu *et al*.^8^ demonstrates that eosinophil activation depends in part on the TGF-β/Smad pathway.^8^ Sotatercept modulates this pathway and has already been studied in haematological disorders such as anaemia, MDS, and myeloma.^9,10^ Although the precise mechanism remains unclear, the sequence of events, the absence of other newly introduced drugs, and the improvement after sotatercept withdrawal and immunosuppression support a plausible drug-related trigger in a predisposed host. Prospective studies should assess whether baseline eosinophil counts and ANCA screening can identify patients at increased risk for ANCA-associated vasculitis before sotatercept initiation.

## Conclusion

This case presents a complex clinical scenario in a patient with PAH undergoing treatment with sotatercept. While no prior history of autoimmune disease was documented, the constellation of findings ultimately fulfilled diagnostic criteria for EGPA. The temporal proximity between the onset of symptoms and the initiation of sotatercept raises questions about a possible immunological trigger in previously subclinical EGPA. Direct causality cannot be established without further mechanistic studies and additional case series.

## Lead author biography


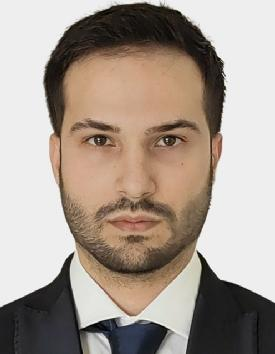


I am Angelo Laconi, a third-year Clinical and Interventional Cardiology resident at Sassari University Hospital, Italy. My research portfolio spans structural heart interventions (TAVI, LAAO), Heart-Failure, Coronary Artery Disease, Cerebrovascular Disease, PAH, AF, and the genetics of cardiac diseases.

## Supplementary Material

ytag025_Supplementary_Data

## Data Availability

The data underlying this article are available in the article and in its online Supplementary material.

## Supplementary material

**Consent:** Written informed consent for publication of this case report and all accompanying images was obtained, in accordance with the CARE guidelines and COPE recommendations. The authors have completed the CARE checklist; the signed checklist is included in the Supplementary material submitted with this manuscript.
