# Supplementary material for: ANCA-associated vasculitis following sotatercept initiation in a patient with heritable pulmonary arterial hypertension and previously silent eosinophilic granulomatosis with polyangiitis: a case report
Source: Eur Heart J Case Rep. 2026 Jan 27;10(2):ytag025. doi: 10.1093/ehjcr/ytag025 (PMC12884410; doi:10.1093/ehjcr/ytag025)
Supplement: ytag025_Supplementary_Data [file ytag025_supplementary_data.docx]

# Supplementary Data Echocardiography follow-up Table

| Date | TAPSE (mm) | sPAP (mmHg) | IT Severity | RV Dilation | Therapy at Time |
| --- | --- | --- | --- | --- | --- |
| 15/06/2016 | 23 | 100 | Mild‑Moderate | Dilated | Macitentan 10 mg; Sildenafil 20 mg ×3; Lasix 25 mg ×2; Canrenone 50 mg |
| 11/07/2016 | 24 | 90 | Mild‑Moderate | Dilated | Continued therapy |
| 14/09/2016 | 24 | 75 | Mild‑Moderate | Dilated | Continued therapy |
| 24/01/2017 | 25 | 65 | Mild‑Moderate | Borderline | Continued therapy |
| 22/02/2017 | 20 | 70 | Severe | Dilated | Indicated for prostanoids; continued therapy |
| 17/10/2017 | 19 | 80 | Mild, High Velocity | Borderline | Riociguat 2.5 mg ×3; macitentan 10 mg |
| 16/10/2018 | 25 | 80 | Mild, High Velocity | Dilated | Selexipag 600 µg/12 h added |
| 14/07/2020 | 22 | 71 | Mild, High Velocity | Dilated | Selexipag 800 µg/12 h; macitentan 10 mg; riociguat 2.5 mg ×3 |
| 23/05/2024 | 15 | 100 | Severe | Markedly dilated | Prostanoid therapy introduced |
| 08/11/2024 | 13 | 77 | Mild, High Velocity | Markedly dilated | Sotatercept initiated |
| 16/01/2025 | 11 | 101 | Severe | Markedly dilated | Rituximab initiated |
| 28/01/2025 | 21 | 97 | Severe‑Massive | Markedly dilated | Continued therapy |

*Supplementary Table 1: Echocardiographic Parameters*

# Right Heart Catheterizations follow-up Table

| Date | PAPm (mmHg) | RAP (mmHg) | Cardiac Index (L/min/m²) | PVR (WU) | Therapy at Time |
| --- | --- | --- | --- | --- | --- |
| 15/06/2016 | 45 | 4 | 1.45 | 12.96 | Macitentan 10 mg; Sildenafil 20 mg ×3; Lasix 25 mg ×2; Canrenone 50 mg |
| 19/10/2016 | 47 | NA | 2.5 | 11.0 | Continued therapy |
| 10/03/2017 | 45 | NA | 2.5 | 6.0 | Switched from sildenafil to riociguat 1 mg TID |
| 04/07/2017 | 36 | NA | 3.23 | 4.7 | Riociguat tittered to 2.5 mg TID in May |
| 16/10/2018 | 41 | NA | 4.56 | 3.9 | Added selexipag 600 µg BID titrated to 800 µg BID |
| 09/03/2023 | 38 | 2 | 4.2 | 4.6 | Follow‑up, clinically stable |
| 23/05/2024 | 47 | NA | 3.08 | 8.0 | Treprostinil started |
| 08/11/2024 | 36 | 4 | 2.88 | 7.1 | Sotatercept added |
| 29/01/2025 | 35 | 5 | 3.22 | 5.59 | Rituximab initiated |

*Supplementary Table 2: Right Heart Catheterization Parameters*

# Laboratory Trend

De‑identified Lab results. One row per visit. Columns and units as in dataset.

| Date | Eos (%) | Eos absolute number(10^3/µL) | WBC (10^3/µL) | Neut (%) | Lymph (%) | Monocytes (%) | Basophils (%) | Creatinine (mg/dl) | Notes |
| --- | --- | --- | --- | --- | --- | --- | --- | --- | --- |
| 2016-08-06 | 3.53 | 0.27 | 7.5 | 49.78 | 40.3 | 5.48 | 0.96 |  |  |
| 2016-09-10 | 5.64 | 0.39 | 6.89 | 50.7 | 36.5 | 6.37 | 0.79 |  |  |
| 2016-11-12 | 3.28 | 0.22 | 6.58 | 46.7 | 42.3 | 6.45 | 1.31 |  |  |
| 2017-03-04 | 3.27 | 0.26 | 8.01 | 55.6 | 34.4 | 5.93 | 0.8 |  |  |
| 2017-04-08 | 5.19 | 0.32 | 6.1 | 39.6 | 49.8 | 4.73 | 0.71 |  |  |
| 2017-05-20 | 4.77 | 0.27 | 5.74 | 44.2 | 44.9 | 4.53 | 1.57 |  |  |
| 2017-07-01 | 9.41 | 0.56 | 5.92 | 41.4 | 42.9 | 5.62 | 0.72 |  |  |
| 2017-08-05 | 9.73 | 0.67 | 6.86 | 33.8 | 50.6 | 4.67 | 1.15 |  |  |
| 2017-09-03 | 8.39 | 0.54 | 6.39 | 42.6 | 43.7 | 4.39 | 0.91 |  |  |
| 2017-11-08 | 11.10 | 0.76 | 6.9 | 40.5 | 41.1 | 6.17 | 1.25 |  | 18/10/2017 pneumonia, clinically resolved 30/10/2017 |
| 2017-11-15 | 11.7 | 0.79 | 6.71 | 35.6 | 45.7 | 5.9 | 1.06 |  |  |
| 2017-12-30 | 5.79 | 0.39 | 6.77 | 39.3 | 48.2 | 5.98 | 0.7 |  |  |
| 2018-03-03 | 6.31 | 0.39 | 6.12 | 48.7 | 39.7 | 4.75 | 0.61 |  |  |
| 2018-04-28 | 3.78 | 0.21 | 5.49 | 43.5 | 46.6 | 6.14 | 0.0 |  |  |
| 2018-05-26 | 7.34 | 0.42 | 5.68 | 41.2 | 44.4 | 5.32 | 1.75 |  |  |
| 2018-07-28 | 4.72 | 0.33 | 6.91 | 40.5 | 48.0 | 5.99 | 0.76 |  |  |
| 2018-10-13 | 5.52 | 0.33 | 6.03 | 39.8 | 49.4 | 4.43 | 0.79 |  |  |
| 2018-12-01 | 6.7 | 0.37 | 5.53 | 46.3 | 40.1 | 4.6 | 1.0 |  |  |
| 2019-01-26 | 7.34 | 0.49 | 6.72 | 41.8 | 44.0 | 5.48 | 1.38 |  |  |
| 2019-04-06 | 8.24 | 0.59 | 7.2 | 50.3 | 37.0 | 4.31 | 0.23 |  |  |
| 2019-05-14 | 6.95 | 0.44 | 6.3 | 46.6 | 40.4 | 4.65 | 1.44 |  |  |
| 2019-06-15 | 5.77 | 0.36 | 6.32 | 41.0 | 46.6 | 5.25 | 1.46 |  |  |
| 2019-08-03 | 7.87 | 0.53 | 6.73 | 46.7 | 40.9 | 4.07 | 0.43 |  |  |
| 2019-09-14 | 6.81 | 0.46 | 6.78 | 47.0 | 40.7 | 4.89 | 0.62 |  |  |
| 2019-11-16 | 5.63 | 0.31 | 5.51 | 42.4 | 45.7 | 5.39 | 0.92 |  |  |
| 2019-12-21 | 8.45 | 0.58 | 6.89 | 40.78 | 44.2 | 5.21 | 1.37 |  |  |
| 2020-01-04 | 9.18 | 0.51 | 5.6 | 38.4 | 46.2 | 5.7 | 0.58 |  |  |
| 2020-02-22 | 4.89 | 0.31 | 6.38 | 47.5 | 42.1 | 4.52 | 0.97 |  |  |
| 2020-11-27 | 5.1 | 0.4 | 7.07 | 46.6 | 41.5 | 4.4 | 0.9 |  | HB ed HCT elevated, ANA 1:320, RA neg ENA/CTD neg |
| 2021-02-16 | 8.3 | 0.5 | 6.38 | 37.0 | 48.5 | 3.9 | 0.8 |  | ANA 1:80, anti CCP neg, ENA neg, JO1 -, B2glic neg, anti-cardiolipine IgG IgM neg, RA - |
| 2021-06-01 | 11.7 | 0.9 | 7.32 | 38.1 | 43.6 | 4.4 | 1.0 |  | IgE elevated 340 U/mL (max value 100 u/mL) |
| 2021-06-23 | 10.6 | 0.8 | 7.49 | 33.8 | 48.7 | 3.7 | 0.8 |  |  |
| 2021-07-23 | 7.12 | 0.53 | 7.46 | 29.0 | 58.0 | 4.79 | 1.06 |  |  |
| 2022-02-08 | 11.5 | 0.8 | 7.04 | 37.8 | 43.4 | 4.8 | 0.9 |  |  |
| 2022-04-20 | 10.9 | 0.9 | 7.79 | 36.9 | 45.3 | 4.5 | 0.9 |  |  |
| 2022-09-10 | 11.5 | 0.77 | 6.7 | 36.65 | 43.8 | 6.62 | 1.33 |  | GPT, GOT gammaGT (2-fold) |
| 2022-10-31 | 26.1 | 2.1 | 8.17 | 25.7 | 41.4 | 4.2 | 0.5 |  |  |
| 2023-02-28 | 13.2 | 1.0 | 7.88 | 35.1 | 44.4 | 4.8 | 0.8 |  |  |
| 2023-03-04 | 4.5 | 0.4 | 7.74 | 59.5 | 29.4 | 4.3 | 0.4 |  | ENA, Jo1, RNP, antiSM, SCL70, Anti-Ro-Anti-La NEGATIVE |
| 2023-08-02 | 11.0 | 0.9 | 7.99 | 50.4 | 31.0 | 5.7 | 0.5 |  |  |
| 2024-01-16 | 9.3 | 0.5 | 5.34 | 40.5 | 42.8 | 4.6 | 0.6 |  |  |
| 2024-09-03 | 8.7 | 0.5 | 5.71 | 49.6 | 32.8 | 6.7 | 0.7 |  |  |
| 2024-09-24 | 8.1 | 0.4 | 5.3 | 44.0 | 39.2 | 5.3 | 1.0 |  | Hospital admission |
| 2024-09-25 | 8.7 | 0.6 | 7.32 | 46.6 | 37.3 | 4.9 | 0.9 |  |  |
| 2024-09-26 | 10.0 | 0.6 | 5.59 | 41.8 | 39.3 | 5.1 | 1.6 |  |  |
| 2024-09-27 | 11.4 | 0.6 | 4.99 | 40.7 | 39.5 | 5.3 | 0.8 |  |  |
| 2024-09-29 | 10.3 | 0.5 | 5.03 | 43.8 | 37.2 | 5.5 | 1.2 |  |  |
| 2024-09-30 | 11.3 | 0.5 | 4.74 | 45.3 | 35.7 | 4.7 | 1.2 |  |  |
| 2024-10-01 | 11.2 | 0.6 | 5.22 | 47.3 | 33.9 | 4.9 | 1.1 |  |  |
| 2024-10-02 | 13.6 | 0.7 | 5.12 | 39.4 | 39.1 | 5.1 | 1.1 |  |  |
| 2024-10-03 | 10.4 | 0.7 | 6.3 | 51.0 | 30.8 | 4.9 | 0.9 |  |  |
| 2024-10-04 | 8.5 | 0.5 | 5.92 | 52.8 | 31.2 | 5.0 | 0.9 |  |  |
| 2024-10-05 | 10.4 | 0.7 | 6.42 | 44.3 | 37.9 | 4.6 | 0.5 |  |  |
| 2024-10-07 | 12.3 | 0.7 | 5.94 | 43.4 | 36.0 | 5.2 | 1.4 |  |  |
| 2024-10-09 | 8.4 | 0.5 | 6.18 | 39.6 | 44.5 | 4.8 | 0.4 |  |  |
| 2024-10-11 | 14.2 | 0.8 | 5.91 | 45.1 | 31.9 | 6.3 | 0.6 |  |  |
| 2024-10-12 | 12.7 | 0.8 | 6.06 | 49.8 | 30.3 | 5.2 | 0.4 |  |  |
| 2024-10-13 | 14.2 | 0.9 | 6.08 | 43.5 | 35.2 | 5.0 | 0.6 |  |  |
| 2024-10-14 | 13.3 | 0.9 | 6.51 | 47.2 | 33.1 | 4.5 | 0.4 |  |  |
| 2024-10-15 | 15.7 | 0.9 | 5.79 | 46.7 | 30.5 | 5.0 | 0.5 |  |  |
| 2024-10-17 | 12.4 | 0.8 | 6.32 | 51.2 | 28.7 | 5.5 | 0.4 |  |  |
| 2024-10-20 | 11.7 | 0.7 | 6.00 | 55.7 | 24.6 | 6.0 | 0.5 |  |  |
| 2024-10-21 | 13.1 | 0.9 | 6.63 | 46.8 | 32.9 | 5.0 | 0.3 |  |  |
| 2024-10-22 | 8.4 | 0.7 | 7.78 | 59.6 | 26.0 | 4.2 | 0.5 |  |  |
| 2024-10-23 | 14.5 | 0.9 | 6.23 | 47.2 | 31.5 | 4.4 | 0.6 |  | SOTATERCEPT initiation |
| 2024-10-24 | 12.6 | 0.9 | 6.81 | 51.6 | 28.7 | 5.0 | 0.7 |  |  |
| 2024-10-25 | 11.0 | 0.9 | 7.78 | 53.6 | 28.6 | 4.3 | 1.0 |  |  |
| 2024-10-26 | 10.0 | 0.8 | 8.26 | 56.4 | 27.3 | 4.5 | 0.6 |  |  |
| 2024-10-27 | 14.3 | 1.1 | 7.49 | 46.9 | 31.8 | 5.1 | 0.3 |  |  |
| 2024-10-28 | 11.5 | 0.8 | 7.14 | 54.3 | 26.2 | 5.5 | 0.7 |  |  |
| 2024-10-29 | 14.0 | 1.1 | 7.81 | 49.0 | 29.6 | 5.1 | 0.6 |  |  |
| 2024-10-31 | 16.9 | 1.2 | 6.86 | 50.8 | 25.6 | 5.0 | 0.4 |  |  |
| 2024-11-05 | 24.7 | 1.7 | 6.92 | 46.8 | 21.3 | 5.4 | 0.5 |  |  |
| 2024-11-08 | 27.7 | 2.1 | 7.51 | 44.2 | 22.1 | 4.2 | 0.4 |  |  |
| 2024-11-09 | 27.1 | 2.0 | 7.53 | 45.5 | 22.4 | 3.5 | 0.4 |  | 2nd dose SOTATERCEPT |
| 2024-11-29 | 58.0 | 9.6 | 16.54 | 23.8 | 14.9 | 2.1 | 0.4 |  | 3rd dose SOTATERCEPT |
| 2024-12-17 | 55.4 | 8.1 | 14.59 | 22.4 | 18.6 | 2.1 | 0.3 |  |  |
| 2025-01-12 | 23.5 | 2.2 | 9.54 | 45.1 | 25.0 | 4.4 | 0.5 |  | Hospital readmission, Vasculitis since 2 weeks |
| 2025-01-13 | 31.0 | 3.2 | 10.19 | 42.2 | 21.2 | 3.7 | 0.4 |  | 14/01 pANCA +, MPO 168IU/mL, cANCA negative |
| 2025-01-15 | 35.1 | 4.1 | 11.63 | 40.3 | 18.8 | 3.9 | 0.3 | 0.88 |  |
| 2025-01-17 | 35.3 | 3.5 | 9.8 | 40.9 | 17.9 | 4.5 | 0.3 | 1.06 |  |
| 2025-01-18 | 4.3 | 0.4 | 10.2 | 39.7 | 48.7 | 4.4 | 0.5 | 1.45 |  |
| 2025-01-19 | 3.4 | 0.5 | 13.31 | 37.1 | 52.9 | 3.5 | 0.4 | 1.81 |  |
| 2025-01-20 | 0.7 | 0.1 | 9.32 | 65.2 | 24.7 | 6.3 | 0.25 | 2.29 | AST 2609uUi/mL, ALT 1076, GammaGT 190, AF 212, LDH 2871 |
| 2025-01-21 | - | - | - | - | - | - | - | 2.4 | BUN 174mg/dL, IgM EBV indeterminate (28.7, positive >40, negative <20) |
| 2025-01-22 | 1.3 | 0.2 | 11.49 | 67.7 | 22.5 | 5.4 | 0.4 | 2.45 | BUN 190 mg/dL, AST 1776, ALT 1090, gammaGT 182, Alcaline phosphatase 205, alfa1antitrip 270 (limite200), IgG and IgM normal range |
| 2025-01-23 | - | - | - | - | - | - | - | 2.26 | 22/01 Metilprednisolone 250 mg |
| 2025-01-24 | - | - | - | - | - | - | - | 1.85 |  |
| 2025-01-25 | 0.3 | 0.0 | 5.26 | 85.0 | 9.8 | 3.8 | 0.0 | 1.34 |  |

*Supplementary Table 3: Laboratory findings with eosinophils trend*

*
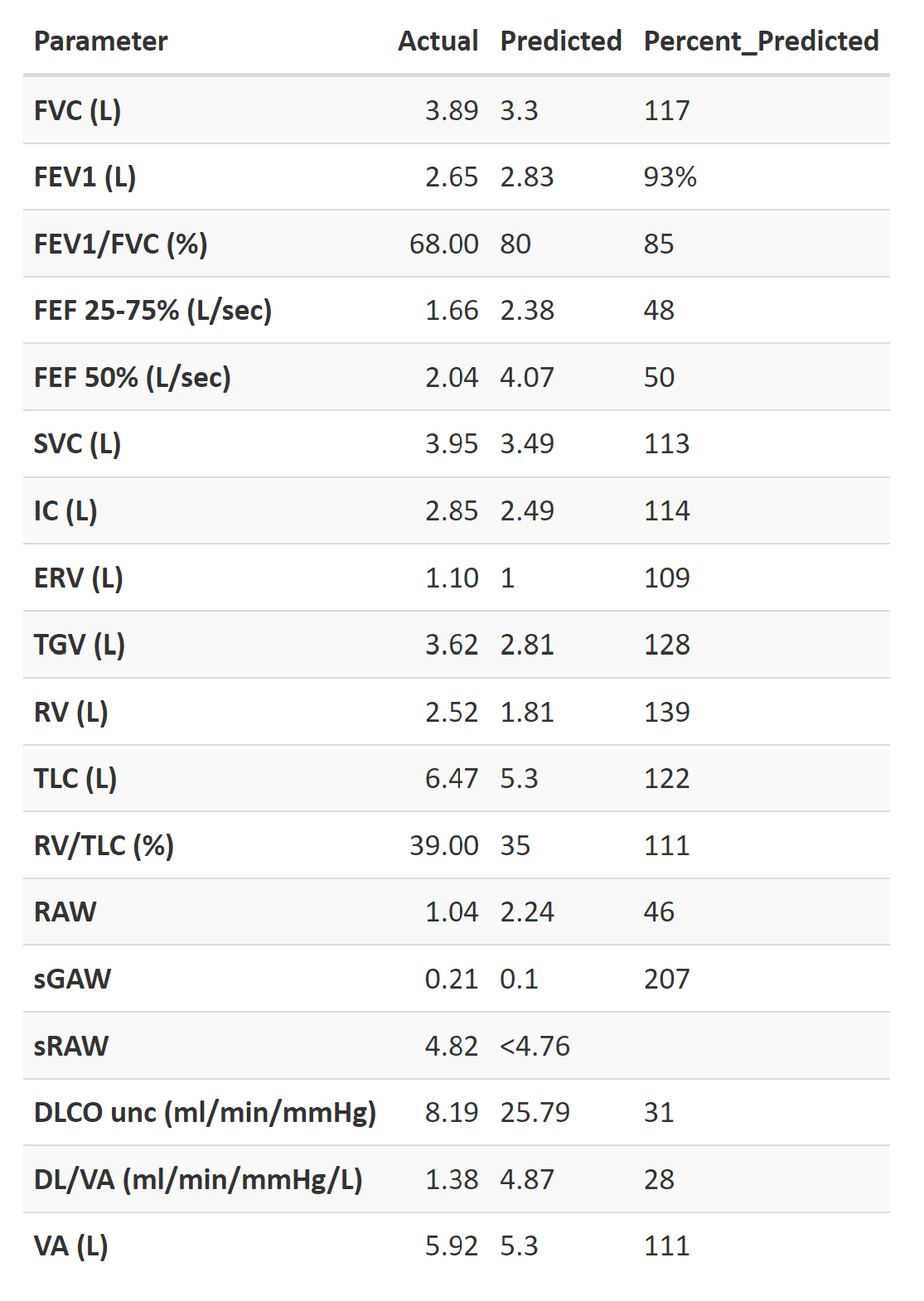
*

*Supplementary figure 1: spirometry at diagnosis*

*
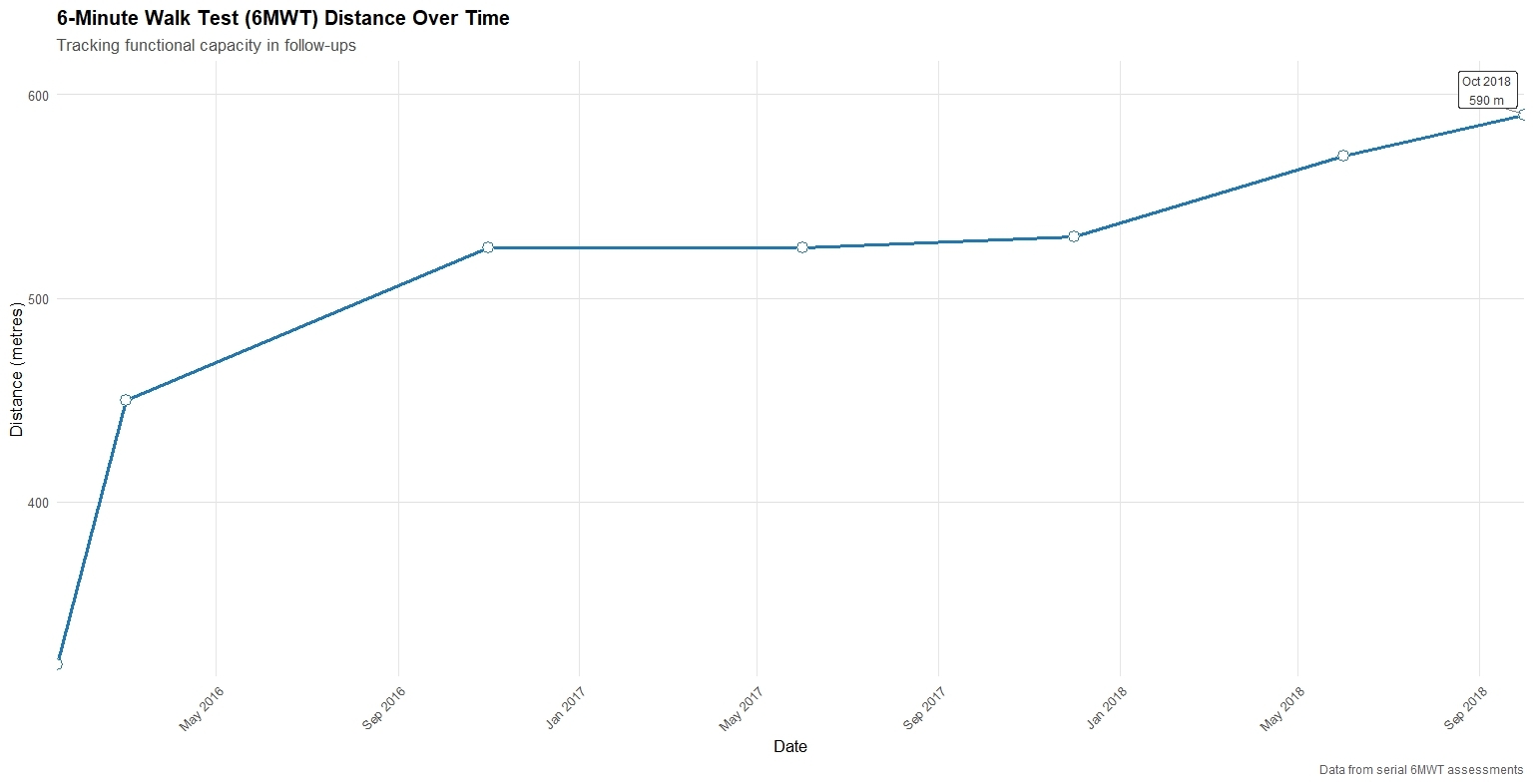
*

*Supplementary figure 2: Functional capacity trend with 6 Minutes Walking Distance*

*
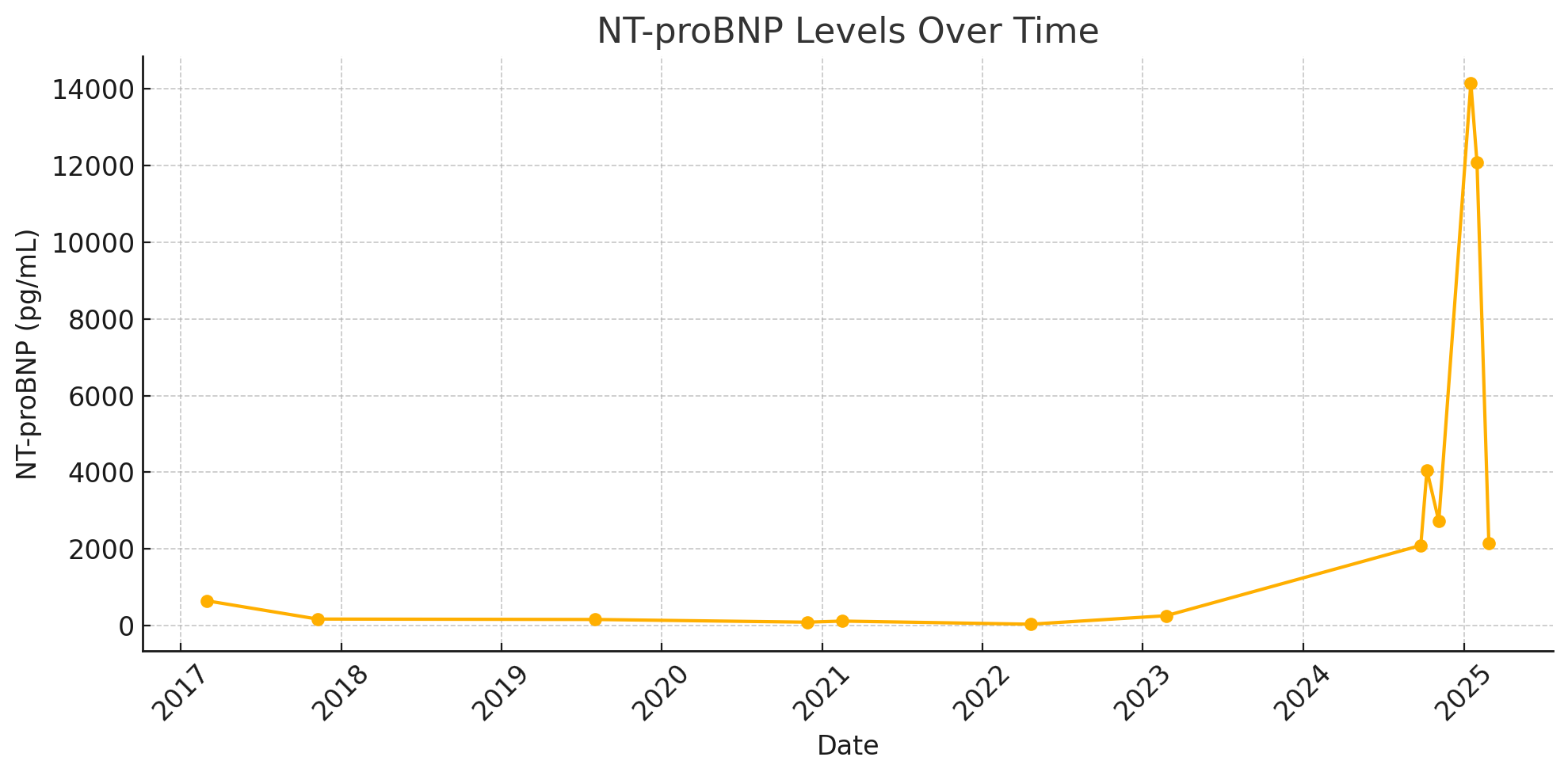
*

*Supplementary figure 3: NT-proBNP trend over time.*
